# Phocine Distemper Virus in Northern Sea Otters in the Pacific Ocean, Alaska, USA

**DOI:** 10.3201/eid1506.090056

**Published:** 2009-06

**Authors:** Tracey Goldstein, Jonna A.K. Mazet, Verena A. Gill, Angela M. Doroff, Kathy A. Burek, John A. Hammond

**Affiliations:** University of California, Davis, California, USA (T. Goldstein, J.A.K. Mazet); US Fish and Wildlife Service, Anchorage, Alaska, USA (V.A. Gill, A.M. Doroff); Alaska Veterinary Pathology Services, Eagle River, Alaska, USA (K.A. Burek); Stanford University School of Medicine, Stanford, California, USA (J.A. Hammond); 1 Current affiliation: Institute for Animal Health, Newbury, UK.

**Keywords:** Viruses, phocine distemper virus, northern sea otters, Alaska, Pacific Ocean, dispatch

## Abstract

Phocine distemper virus (PDV) has caused 2 epidemics in harbor seals in the Atlantic Ocean but had never been identified in any Pacific Ocean species. We found that northern sea otters in Alaska are infected with PDV, which has created a disease threat to several sympatric and decreasing Pacific marine mammals.

In northern Europe, phocine distemper virus (PDV) caused 2 epidemics that resulted in 23,000 harbor seal deaths in 1988 and >30,000 deaths in 2002 (*1*). PDV has also been associated with seal deaths on the eastern coast of the United States and Canada, which shows the persistent threat of this virus to Atlantic marine mammal populations (*2*). Serologic surveys before 2000 indicated that Pacific marine mammals had not been exposed to PDV (*3*,*4*), and this virus had never been identified as the cause of illness or death in the North Pacific Ocean. In this region, specifically in Alaska, northern sea otters (*Enhydra lutris kenyoni*) are one of many species that have had population decreases since the 1980s. Steller sea lion (*Eumetopias jubatus*), northern fur seal (*Callorhinus ursinus*), and most recently, harbor seal (*Phoca vitulina*) populations have all decreased (*4*–*6*).

## The Study

**Figure 1:**
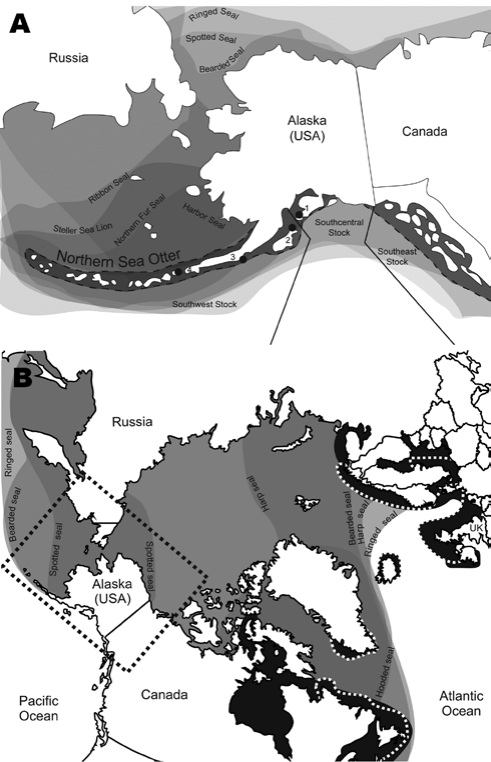
Distribution of Arctic and sub-Arctic pinnipeds in relation to Arctic ice coverage representing a unique area where distribution ranges of multiple seal species overlap (*7*,*8*). A) North Pacific Ocean region showing the range of the northern sea otter (*Enhydra lutris kenyoni*) in Alaska, its population stock delineations, and sample collection locations for the study. 1, Kachemak Bay; 2, Kodiak Archipelago; 3, South Alaska Peninsula; 4, Fox Island; seal species ranges overlap. This overlap indicates potential for phocine distemper virus disease transmission among Arctic and sub-Arctic pinniped species in this highly productive region. B) Circumpolar Arctic region showing species overlap among Arctic pinnipeds and the potential for disease transmission from the Atlantic Ocean through the Arctic Ocean to Alaska (outlined) by migrating seal species. The black areas indicate ranges of Atlantic harbor and gray seals; the areas exclusive to gray seal are bordered with a broken line. The boxed region corresponds to the Arctic region containing sea otter populations shown in panel A.

In 2004 and 2005, strong serologic evidence of exposure to a PDV-like morbillivirus was obtained by serum neutralization for ≈40% (30/77) of live captured sea otters sampled in the eastern Aleutian Islands (Fox Island, South Alaska Peninsula) and Kodiak Archipelago (T. Goldstein et al., unpub. data) (Figure 1, panel A, southwest stock). These captures were part of an investigation into potential causes of a precipitous decrease in the population that resulted in a US Endangered Species Act listing. Although northern sea otters are found along the coast of Alaska, Canada, and Washington and in the Aleutian Islands, only the southwest stock in Alaska has been decreasing (*9*) (Figure 1, panel A). As little as 50% of the southwest stock remains since the 1980s, and the Aleutian Archipelago population decreased from ≈74,000 to 8,742 by 2000.

In 2006, the US Working Group on Marine Mammal Unusual Mortality Events declared an unusual mortality event for northern sea otters; large numbers of deaths were documented in southcentral Alaska adjacent to the threatened southwest stock (V. Gill, unpub. data) (Figure 1, panel A). Necropsies showed a high prevalence of valvular endocarditis (43%) and septicemia in mature adults associated with various strains of *Streptococcus infantarius* subsp. *coli* (*S*. *bovis/equinus* complex) and inconsistent intracytoplasmic inclusions were present. However, a primary site of bacterial infection could not be identified in most infected animals, despite this high prevalence of lesions. In humans, *S*. *bovis* is a major cause of valvular endocarditis and is often associated with preexisting pathologic changes of the colon, underlying disease, and immunosuppression (*10*). This disease is often sporadic and secondary to chronic recurrent bacterial seeding from a primary site of infection or secondary to heart valve abnormalities. The lack of underlying bacterial infection or heart valve defects indicated the presence of a primary immunosuppressive viral infection.

To further investigate serologic evidence and necropsy findings, we looked for morbilliviral nucleic acid in nasal swabs archived from live otters and in tissue (brain, lung, lymph node) from 9 stranded carcasses from Kachemak Bay (southcentral stock, Figure 1, panel A) examined during 2005–2008. Total RNA was extracted by using Tri Reagent (Sigma, St. Louis, MO, USA) and complimentary DNA was transcribed by using Superscript III (Invitrogen, Carlsbad, CA, USA) with random nonamers. A heminested PCR was performed with universal morbillivirus primers and a PDV-specific primer for the phosphoprotein gene (*11*). Products of the expected size were sequenced.

**Figure 2:**
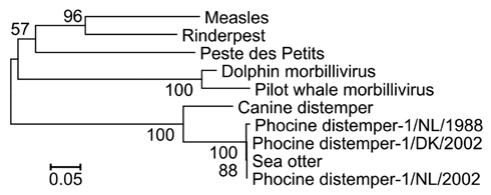
Neighbor-joining bootstrap tree (1,000 replicates, pairwise deletion comparisons, Tamura-Nei model) shows that morbillivirus fragments isolated from northern sea otters are identical to those of the 2002 PDV isolates. All known corresponding phosphoprotein gene fragments from morbilliviruses (Technical Appendix) were compared by using Molecular Evolutionary Genetics Analysis software version 3.1 (www.megasoftware.net/mega.html). Scale bar indicates number of nucleotide substitutions per site.

Morbilliviral nucleic acid was amplified from 8 nasal swabs from live otters (10%, 8/77) and from lung, lymph node, or brain from 3 dead otters. Sequence analysis identified a PDV fragment identical to that of the isolate from the 2002 outbreak in northern Europe. This PDV fragment differed from the 1988 isolate at 2 nucleotide positions (Technical Appendix; Figure 2). The PDV-positive nasal swabs were from 5 juveniles and 3 adults, 7 from the Kodiak Archipelago and 1 from the Eastern Aleutians in 2004 and 2005. Seven of these 8 otters were also positive for antibodies to PDV by serum neutralization. The dead PDV-positive otters were 2 adults and 1 juvenile from Kachemak Bay sampled during 2005–2007. The cause of death in these animals included meningoencephalitis and/or sepsis with or without valvular endocarditis. This finding mirrors the secondary bacterial infections characteristic of infected and immunosuppressed European harbor seals during PDV epidemics (*1*).

## Conclusions

These results demonstrate that PDV has been introduced to the North Pacific Ocean since 2000. All Pacific marine mammal species are now at risk for phocine distemper–induced population decreases. Although additional work is needed to determine if PDV has played a role in the decrease in the sea otter population, its association with lesions in carcasses, especially in animals that have died of bacterial infections, suggests it may contribute to ongoing deaths. Viral nucleic acid in nasal swabs from free-ranging, live-captured otters confirms viral shedding. Therefore, otters are capable of transmitting PDV to conspecifics and other species.

Because the PDV fragment isolated from Alaskan otters is identical to that of the 2002 Atlantic isolate, this virus was likely transmitted to the North Pacific Ocean after the 2002 European epidemic, although it is remotely possible that it may have originated in the North Pacific Ocean during 2000–2002. Several ranges of seal species overlap across the Atlantic and Arctic Oceans (Figure 1, panel B). Arctic and sub-Arctic migrating seals have also been suggested to be carriers of PDV (*1*). In the Atlantic Ocean, gray seals (*Halichoerus grypus*) are vectors of PDV that enable spread of disease to harbor seal populations and provide contact between North Sea and Arctic Ocean species (*12*) (Figure 1, panel B). Although PDV vector species are largely unknown, the close phylogenetic relationship and geographic range of susceptible seals with other seal species makes this intraspecies contact the likely method of transmission through the Arctic to the Pacific Ocean. Now that PDV is in the Pacific Ocean, the diversity and abundance of seal and sea lion species creates the potential for viral transmission (Figure 1).

Serologic evidence indicates that the 1988 Atlantic PDV virus did not reach the Arctic or Pacific regions of Alaska. The decrease in sea ice during the 14 years between these epidemics may have affected movement of Arctic seal populations (Technical Appendix Figure 2). This reduction was even more pronounced in 2004 and 2005, years in which PDV was confirmed to have infected sea otters (Technical Appendix). Ice coverage is at its lowest level during August and September (*14*). In 1988 and 2002, the PDV epidemic had reached gray and harbor seal populations in the North Sea and Norwegian Sea by August. This sea ice reduction may have altered seal haul-out and migration patterns, resulting in contact between Atlantic, Arctic, and Pacific Ocean species that was not possible in 1988 and the few years afterwards.

Now that PDV has been found in the Pacific Ocean, its role in population decreases and future deaths among currently uninfected species of marine mammals in Alaska must be assessed. A subspecies of the susceptible Atlantic harbor seal, the Pacific harbor seal is potentially vulnerable to PDV, and with a range from Alaska and along the West coast of the United States, they have enormous potential to spread the virus. Additionally, because terrestrial and marine Arctic species from Canada have previously been exposed to PDV, the risk for predatory and scavenging North Pacific Ocean carnivore species must not be overlooked (*15*). All seal species in the Arctic and Pacific Oceans are threatened, especially those with limited numbers, and epidemic management strategies must be in place to protect critically small populations.

## Supplementary Material

Technical Appendix Phocine Distemper Virus in Northern Sea Otters in the Pacific Ocean, Alaska, USA

## References

[1] Harkonen T, Dietz R, Reijnders P, Teilmann J, Harding K, Hall A, The 1988 and 2002 phocine distemper virus epidemics in European harbour seals. Dis Aquat Organ. 2006;68:115–30. 10.3354/dao068115 16532603

[2] Duignan PJ, Sadove S, Saliki JT, Geraci JR. Phocine distemper in harbor seals (*Phoca vitulina*) from Long Island, New York. J Wildl Dis. 1993;29:465–9. 8355350 10.7589/0090-3558-29.3.465

[3] Zarnke RL, Saliki JT, Macmillan AP, Brew SD, Dawson CE, Ver Hoef JM, Serologic survey for *Brucella* spp., phocid herpesvirus-1, phocid herpesvirus-2, and phocine distemper virus in harbor seals from Alaska, 1976–1999. J Wildl Dis. 2006;42:290–300. 16870851 10.7589/0090-3558-42.2.290

[4] Burek KA, Gulland FMD, Sheffield G, Beckmen KB, Keyes E, Spraker TR, Infectious disease and the decline of Steller sea lions (*Eumetopias jubatu*s) in Alaska, USA: insights from serologic data. J Wildl Dis. 2005;41:512–24. 16244061 10.7589/0090-3558-41.3.512

[5] York AE. Status, biology, and ecology of fur seals. In: Proceedings of an International Symposium and Workshop. Croxall JP, Gentry RL, editors. Seattle, Washington. Washington: National Oceanic Atmospheric Administration. Tech Rep NMFS 51; 1987. p. 9–21.

[6] Small RJ, Boveng PL, Byrd VG, Withrow DE. Harbor seal population decline in the Aleutian Archipelago. Mar Mamm Sci. 2008;24:845–63.

[7] US Fish and Wildlife Service. Stock assessment for sea otters (*Enhydra lutris*): Southwest Alaska stock. In: Marine mammal protection act stock assessment report 8. Washington: The Service; 2002.

[8] Angliss RP, Outlaw RB. Alaska marine mammal stock assessments. US Department of Commerce: NOAA Technical Memos, NMFS-TM-AFSC-168 (2007) and NMFS-TM-AFSC 180. Washington: The Department; 2008.

[9] Doroff AM, Estes JA, Tinker MT, Burn DM, Evans JA. Sea otter population declines in the Aleutian Archipelago. J Mammal. 2003;84:56–64. 10.1644/1545-1542(2003)084<0055:SOPDIT>2.0.CO;2

[10] Mylonakis E, Calderwood SB. Infective endocarditis in adults. N Engl J Med. 2001;345:1318–30. 10.1056/NEJMra010082 11794152

[11] Barrett T, Visser KG, Mamaev L, Goatley L, van Bressum M-F, Osterhaus AD. Dolphin and porpoise morbilliviruses are genetically distinct from phocine distemper virus. Virology. 1993;193:1010–2. 10.1006/viro.1993.1217 8460473

[12] Hammond JA, Pomeroy PP, Hall AJ, Smith VJ. Identification and real-time PCR quantification of phocine distemper virus from two colonies of Scottish grey seals in 2002. J Gen Virol. 2005;86:2563–7. 10.1099/vir.0.80962-0 16099915

[13] National Snow and Ice Data Center. Sea ice index [cited 2009 Mar 18]. Available from http://nsidc.org/data/seaice_index

[14] Lindsay RW, Zhang J. The thinning of Arctic sea ice, 1988–2003: have we passed a tipping point? J Clim. 2005;18:4879–94. 10.1175/JCLI3587.1

[15] Philippa JD, Leighton FA, Daoust PY, Nielsen O, Pagliarulo M, Schwantje H, Antibodies to selected pathogens in free-ranging terrestrial carnivores and marine mammals in Canada. Vet Rec. 2004;155:135–40. 15338705 10.1136/vr.155.5.135

