# Supplementary material for: Phocine Distemper Virus in Northern Sea Otters in the Pacific Ocean, Alaska, USA
Source: Emerg Infect Dis. 2009 Jun;15(6):925–7. doi: 10.3201/eid1506.090056 (PMC2727316; doi:10.3201/eid1506.090056)
Supplement: Technical Appendix — Phocine Distemper Virus in Northern Sea Otters in the Pacific Ocean, Alaska, USA [file 09-0056_Techapp-s1.pdf]

Phocine Distemper Virus in Northern Sea Otters in the Pacific Ocean, Alaska, USA

Technical Appendix

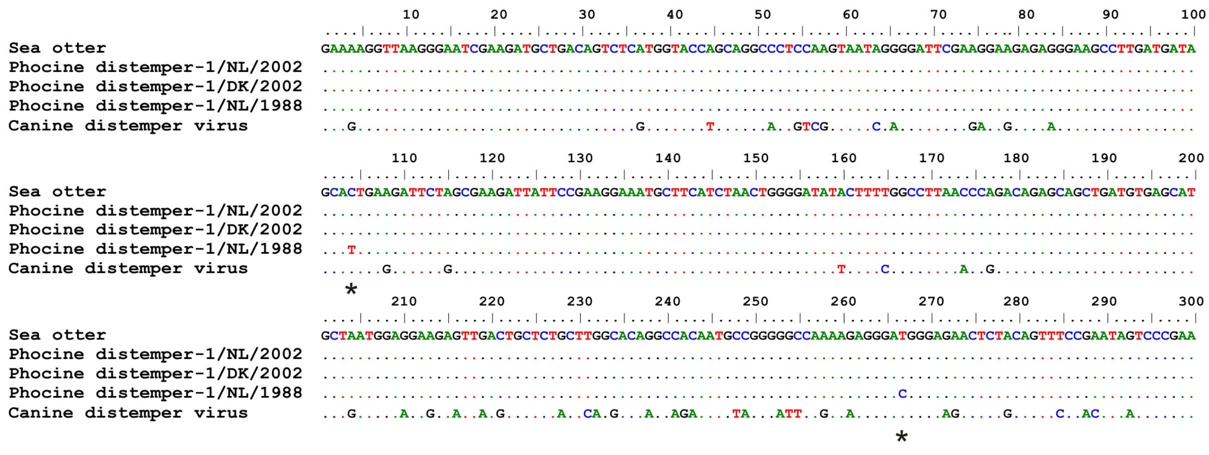

Technical Appendix Figure 1. Nucleotide sequence alignment of the morbillivirus phosphoprotein gene fragment isolated from northern sea otters with the corresponding region from the known phocine distemper virus (PDV) isolates and closely related canine distemper virus. Dots indicate residues identical to those in the sea otter fragment, and asterisks indicate residues that differ between the 1988 and 2002 PDV isolates. Alignment was produced manually by using BioEdit version 7.0.9 (Bioedit Sequence Alignment Editor 1997–2007; T.A. Hall Software, Raleigh, NC, USA).

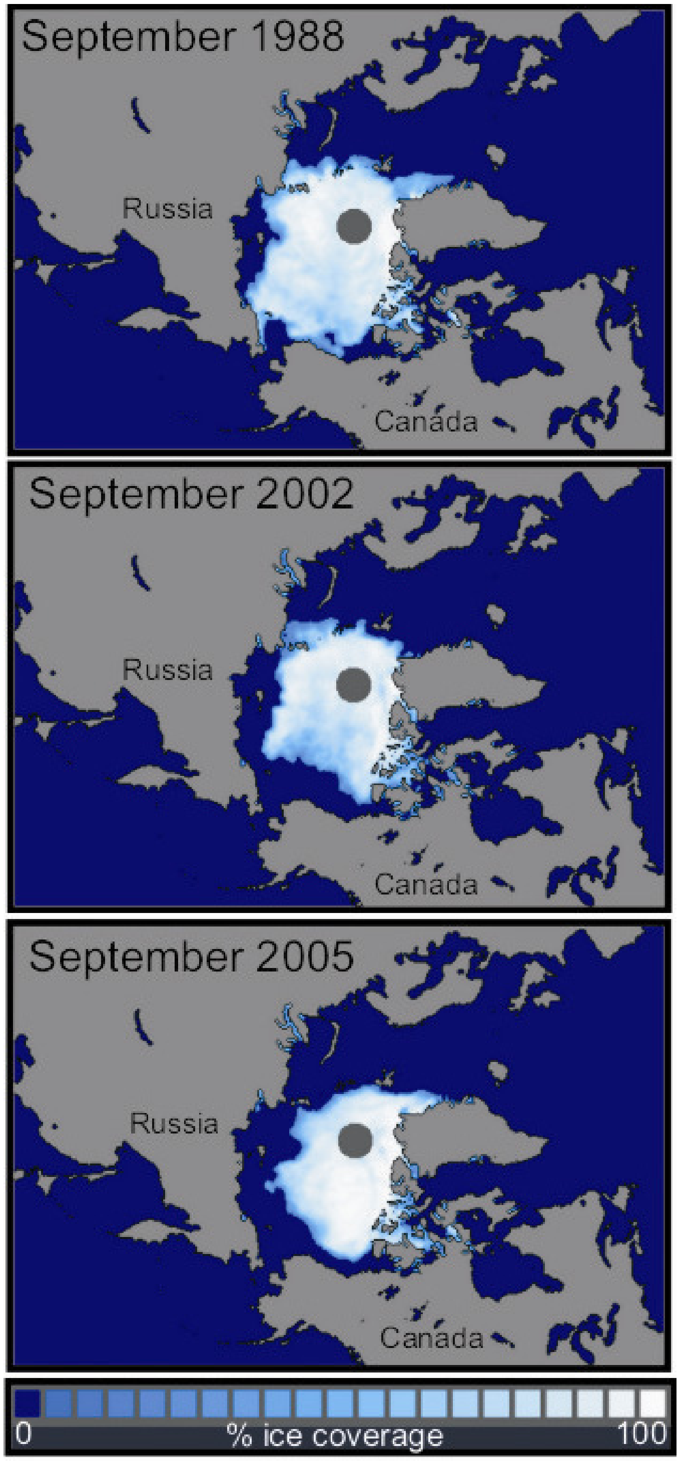

Technical Appendix Figure 2. Reduction of September Arctic ice coverage observed by satellite imagery during 1988–2005, the period between the first phocine distemper virus (PDV) outbreak in the Atlantic Ocean and the latest PDV-positive sea otter nasal swabs. Maps were generated with the Sea Ice Index from the National Snow and Ice Data Center (*13*).
